# Integrated Analys of High‐Fat Challenge‐Induced Changes in Blood Cell Whole‐Genome Gene Expression

**DOI:** 10.1002/mnfr.201900101

**Published:** 2019-09-30

**Authors:** Juri C. Matualatupauw, Colm O'Grada, Maria F. Hughes, Helen M. Roche, Lydia A. Afman, Jildau Bouwman

**Affiliations:** ^1^ Division of Human Nutrition Wageningen University 6700 EV Wageningen The Netherlands; ^2^ Microbiology and Systems Biology TNO 3700 AJ Zeist The Netherlands; ^3^ Nutrigenomics Research Group UCD Conway Institute of Biomolecular and Biomedical Research University College Dublin Dublin 4 D04 N2E5 Ireland

**Keywords:** bioinformatics, high‐fat challenge, microarrays, nutrigenomics, phenotypic flexibility, saturated fatty acids

## Abstract

**Scope:**

Several studies have examined the whole‐genome gene expression response in blood cells to high‐fat challenges with differing results. The study aims to identify consistently up‐ or downregulated genes and pathways in response to a high‐fat challenge using several integration methods.

**Methods and results:**

Three studies measuring the gene expression response to a high‐fat challenge in white blood cells are evaluated for common trends using several integration methods. Overlap in differentially expressed genes between separate studies is examined, *p*‐values of each separate study are combined, and data are analyzed as one merged dataset. Differentially expressed genes and pathways are compared between these methods. Selecting genes differentially expressed in the three separate studies result in 67 differentially expressed genes, primarily involved in circadian pathways. Using the Fishers *p*‐value method and a merged dataset analysis, changes in 1097 and 1182 genes, respectively, are observed. The upregulated genes upon a high‐fat challenge are related to inflammation, whereas downregulated genes are related to unfolded protein response, protein processing, cholesterol biosynthesis, and translation.

**Conclusion:**

A general gene expression response to a high‐fat challenge is identified. Compared to separate analyses, integrated analysis provides added value for the discovery of a consistent gene expression response.

## Graphical abstract

An integrated analysis on microarray datasets from several high‐fat challenge intervention studies is performed. Several integration methods to identify changes in *PBMC* gene expression upon a high‐fat challenge are used. This integrated analysis is shown to provide added value for the discovery of consistently differentially expressed genes and pathways compared to separate analysis of the studies.

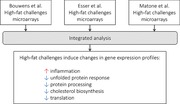

## 1 Introduction

Metabolic challenge tests are performed in nutritional research to magnify the small effects of nutrition on health.^1^ In addition, an individual's response to such a challenge is considered a marker of phenotypic flexibility and reflect the individual's health status. A type of metabolic challenge test that is frequently used in nutrition studies is the high‐fat challenge, in which subjects consume a high‐fat load containing 50–100 g of fat and may contain different type of fat. These high‐fat challenges have been shown to not only affect postprandial lipid metabolism, but also other processes, such as inflammation, stress response, vascular health, and glucose metabolism.^2^

A good source of cells to study the abovementioned processes are peripheral blood mononuclear cells (PBMCs), which have proven useful for the determination of the effects of nutritional interventions on gene expression profiles.^3^, ^4^, ^5^, ^6^ These cells are a subset of circulating white blood cells, which are straightforward to collect during human intervention studies as they can be easily isolated from blood. Several studies have examined the effects of acute high‐fat challenges on PBMC whole genome gene expression by comparing postprandial gene expression profile responses between challenges with different nutritional compositions. Bouwens et al.^7^ found that a high‐SFA challenge induced expression of liver X receptor signaling genes, whereas a high‐PUFA challenge caused a downregulation of these genes. Matone et al.^8^ studied the effects of a high‐MUFA challenge and observed increased expression of inflammatory genes in several pathways, including Toll‐like receptors, interleukins, tumor necrosis factor, and nuclear factor κB‐related genes. Esser et al.^9^ found that a high‐SFA challenge decreased expression of cholesterol biosynthesis and uptake genes and increased cholesterol efflux genes compared to a high‐MUFA challenge that increased expression of inflammatory genes and peroxisome proliferator‐activated receptor (PPAR)‐α target genes involved in β‐oxidation. From the previous, it is clear that the changes in gene expression profiles described in these studies are quite diverse. Therefore, the question is raised if a general response to a high‐fat challenge, independent of fatty acid composition, can be characterized.

In this manuscript, our first aim was to perform and compare different methods of combining the results of the three abovementioned studies and data of an additional study that examined blood cell gene expression response to high‐fat challenges. Second, using this cross‐study integrated analysis, our goal was to identify consistently up or downregulated genes and pathways in response to a high‐fat challenge. Ultimately, this may help to increase our understanding of how the body responds to a high‐fat load and may lead to a marker of health.

## 2 Experimental Section

### 2.1 Study Design

The studies by Bouwens et al., Matone et al., and Esser et al. were previously published and data is publicly available^7^, ^8^, ^9^ (GSE13466, GSE56609, and GSE53232 at Gene Expression Omnibus (https://www.ncbi.nlm.nih.gov/geo)). These studies used single‐channel Affymetrix microarrays to perform gene expression measurements, whereas the fourth study (Fat_challenge_tests) used llumina HumanHt12‐v4 Expression Beadchips. Data for this study is available under GSE124534 and the design and clinical parameters can be found in the Phenotype Database (https://dashin.eu/interventionstudies/9218_Fat_challenge_tests). The studies by Bouwens et al. and Esser et al. applied a crossover design to examine high‐SFA versus high‐MUFA and high‐PUFA challenges, respectively. To decrease heterogeneity between studies, we elected to analyze only the high‐SFA challenges from both of these studies in this integrated analysis. Moreover, of the Fat_challenge_tests study (GSE124534), we only used the high‐fat challenge data that were acquired before the 4‐week high‐fat high‐calorie diet intervention.^10^ In all studies, gene expression measurements were performed before and after the high‐fat challenges. All analyses are performed on the measured change in gene expression. The designs of the four studies are summarized in **Table** 1.

**Table 1:** Summary of the four studies included in the analysis

Study	Subjects	Fat composition	Time of postprandial measurement	Microarray platform	Entrez genes on microarray	Filter criteria	Entrez genes expressed
Bouwens et al. (2010)^7^	21 lean, young men	39 g SFA 14 g MUFA 1 g PUFA	6 h	Affymetrix NuGO Hs1a520180	17 359	Universal exPression Code > 0.5 in > 10 samples	8779
Matone et al. (2015)^8^	33 men and women, ranging in age and BMI	7 g SFA 31 g MUFA 16 g PUFA	4 h	Affymetrix Human Gene 1.0 ST	19 624	Universal exPression Code > 0.5 in > 16 samples	10 352
Esser et al. (2016)^9^	17 lean and 15 obese middle‐age men	51 g SFA 37 g MUFA 6 g PUFA	4 h	Affymetrix Human Gene 1.1 ST	19 621	Universal exPression Code > 0.5 in > 16 samples	9440
Fat_challenge_tests	Eight healthy and eight metabolic syndrome men	66 g SFA 23 g MUFA 3 g PUFA	6 h	Illumina HumanHt12‐v4 Expression Beadchip	30 500	Expression P value < 0.01 in > 4 arrays	10 754

### 2.2 Preprocessing

Preprocessing was done for each study separately. For the studies using Affymetrix microarrays, raw CEL files were normalized using the Robust Multi‐array Average algorithm,^11^ as implemented in the *oligo* R‐package.^12^ A custom annotation was used based on reorganized oligonucleotide probes, which combines all individual probes for a gene (brainarray CDF files (ENTREZG v20)). Low expressing probes genes were filtered out using Universal exPression Codes with a 0.5 cut‐off, corresponding to a 50% likelihood that a gene is expressed.^13^

For the Fat_challenge_tests study, probe expression values were calculated using the neqc algorithm from the *limma* package.^14^ Low expressing probes were filtered by selecting only the probes with a detection *p*‐value < 0.01 in at least five arrays for further analysis. To end up with only one value per Entrez gene, the probe with the highest variance was selected when multiple probes were present for a gene, as variance‐filtering has been shown to increase statistical power.^15^

**Figure 1:**
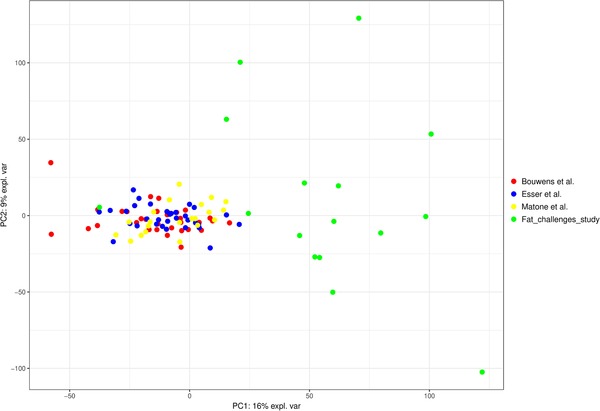
Principal component analysis of the log_2_‐ratios (after intervention/before intervention) of the four studies. Every dot represents one subject.

**Figure 2:**
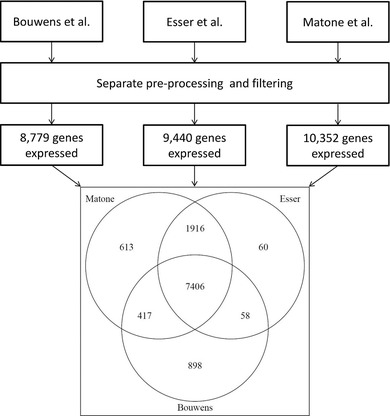
Gene selection workflow.

After filtering, only the genes present in all datasets, were retained for further analysis. Initial analysis showed that the Fat_challenge_tests study showed a large batch effect when examining per subject log_2_‐ratio (**Figure** 1). Therefore, it was decided to exclude this study from further analysis. The three remaining studies by Bouwens et al., Matone et al., and Esser et al. had an overlap of 7406 genes, which were used for further analysis. The workflow is summarized in **Figure** 2.

### 2.3 Differentially Expressed Genes

First, log_2_‐ratios per subject between the intensities before and after the challenge were calculated. These log_2_‐ratios were used for further analysis using the Linear Models for Microarray data (*limma)* R‐package. To identify differentially expressed genes, empirical Bayes moderated *t*‐tests as implemented in *limma* were performed in each dataset separately as well as in the combined dataset of all studies. *p*‐Values were adjusted using Benjamini and Hochberg false discovery rate^16^ and a *Q*‐value < 0.05 was considered significant.

In addition to the *p*‐values per study and the *p*‐values of the combined dataset, Fisher's combined probability test was also used to pool *p*‐values from the three separate studies. The *MetaDE* R‐package was used to calculate a test statistic (Chi^2^) which follows a chi distribution, as well as *p*‐ and false discovery rate (FDR)‐values for each gene.^17^ An FDR *Q* < 0.05 was used as significance cutoff, indicating that the corresponding gene was differentially expressed in at least one study.

### 2.4 Pathway Analysis

For all pathway analyses, a list of gene sets was used that was derived by combining all gene sets taken from the Biocarta, KEGG, Reactome, and Wikipathways databases. Gene set enrichment analysis (GSEA; http://www.broad.mit.edu/gsea) was performed.^18^ Genes were ranked based on the moderated *t*‐statistic and analyzed for over‐ or underrepresentation in the gene sets. GSEA was performed in each separate study as well as in the combined dataset. Gene sets with an FDR *q* < 0.25 were considered significantly enriched.

## 3 Results

In this study, we reanalyzed data of four separate studies of which the study designs are described in Table 1. Baseline characteristics of the study populations can be found in **Table** 2. Age was different between all studies, weight and BMI were both lower in the Bouwens et al. study and height was significantly higher in the Bouwens et al. and the Fat_challenge_tests study. To examine the presence of a potential batch effect between studies, we performed principal component analysis (PCA) on the per subject change in gene expression depicted as log_2_‐ratios (Figure 1). The Fat_challenge_tests study showed a separation from the other three studies, indicating the presence of large differences in gene expression changes between this study and the others. Therefore, this study was excluded for further analyses and the analysis was continued using the Bouwens et al., Matone et al., and Esser et al. studies only.

**Table 2:** Baseline characteristics of the four study populations

	Bouwens et al.^7^	Matone et al.^8^	Esser et al.^9^	Fat_challenge_tests	*p*‐Value
Age [years]	21 ± 3^a^	37.3 ± 13^b^	62 ± 5^c^	46 ± 7^d^	<0.001
Weight [kg]	74.4 ± 8.1^a^	83.0 ± 18.0^b^	89.0 ± 18.0^b^	86.8 ± 13.0^b^	0.009
Height [m]	1.84 ± 0.06^a^	1.75 ± 0.09^b^	1.78 ± 0.07^bc^	1.81 ± 0.07^ac^	0.001
BMI [kg m^–2^]	22.1 ± 2.0^a^	27.0 ± 6.1^b^	27.9 ± 4.9^b^	26.4 ± 3.2^b^	<0.001

Data are presented as mean ± SD. Differences between groups were determined using one‐way ANOVA and corresponding *p*‐values are shown. Different letters indicate differences between groups, as determined using LSD post hoc tests.

**Figure 3:**
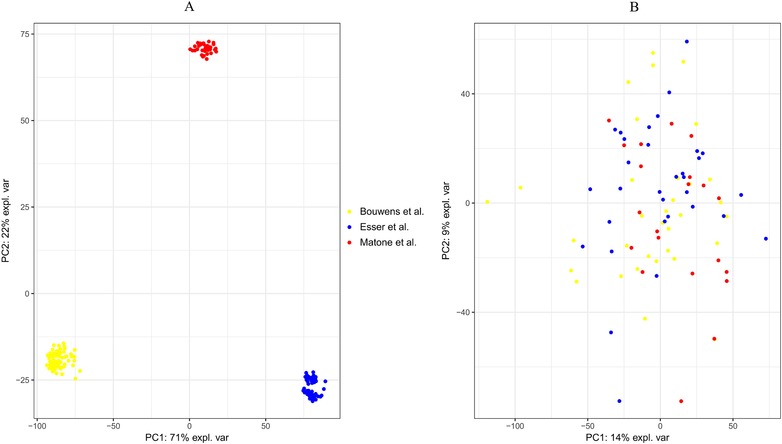
A) Principal component analysis of log_2_‐intensity values of all individual samples from the three studies. B) Principal component analysis of log_2_‐ratios of the response upon the high‐fat challenge (after intervention/before intervention) of all subjects from the three studies.

In Figure 2, the number of genes after filtering out non‐expressed genes per data set/study and the overlapping genes between datasets are shown. Combining the three datasets resulted in 7406 genes that were expressed in all datasets and these were used for further analysis. To examine variation within and between the datasets, we performed a PCA on the normalized log_2_ intensity values. This showed a clear separation between datasets (**Figure** 3A), reflecting between‐study batch effects. When the PCA was performed using per subject log_2_‐ratios of the postprandial effects, datasets were found to be intermixed (Figure 3B). We continued our analyses using these log_2_‐ratios.

### 3.1 Differentially Expressed Genes

**Figure 4:**
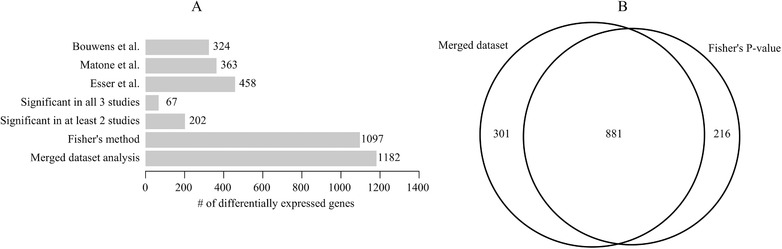
A) Number of significantly differentially expressed genes identified in each separate dataset and in the integrated analysis of the datasets using different methods. Overlap in genes between all results are shown in Figure S1, Supporting Information. B) Venn diagram of overlap between Fishers method and the merged dataset analysis.

**Figure 5:**
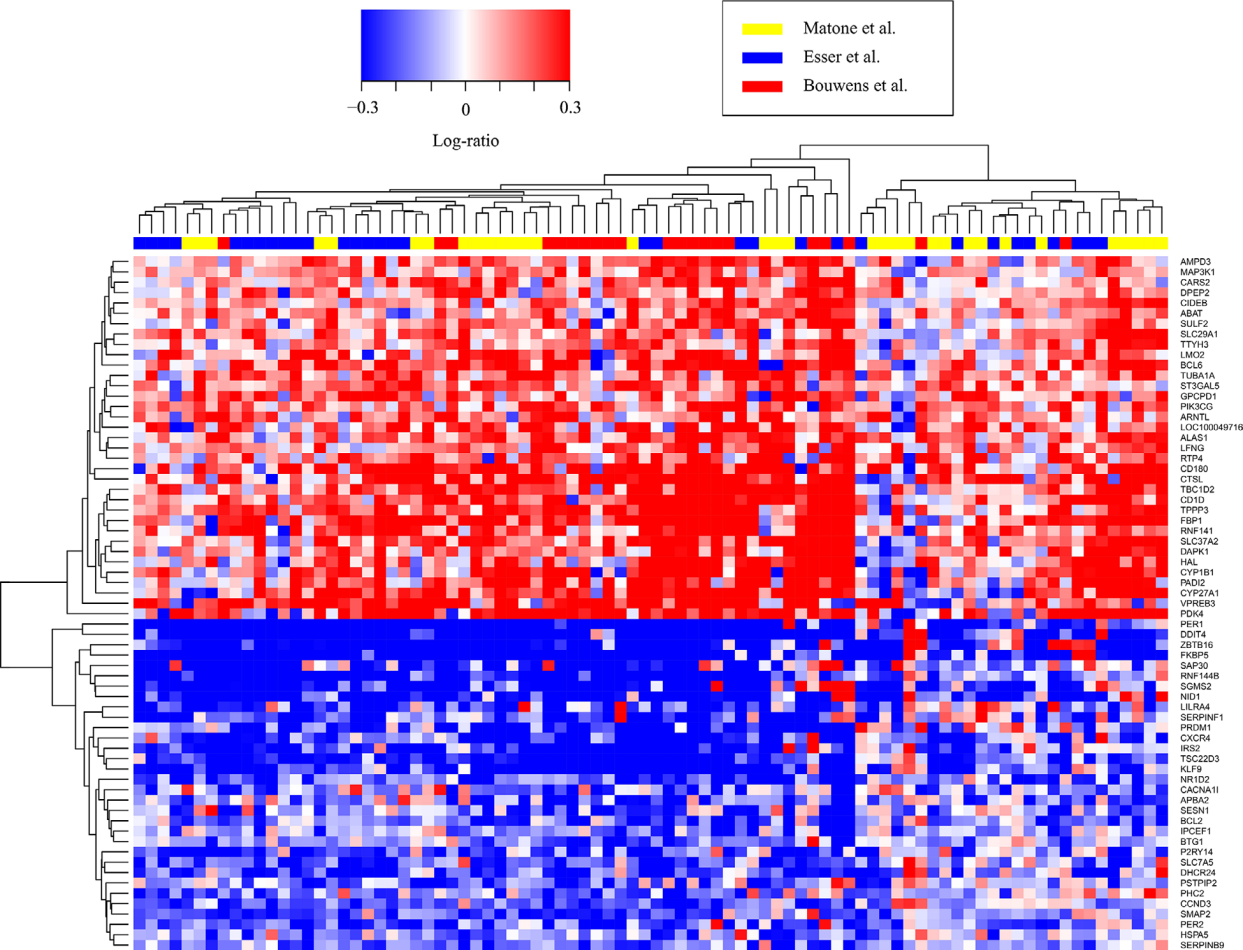
Heatmap depicting individual gene expression changes by high‐fat challenges of the 67 genes that are differentially expressed in all studies (FDR *Q* < 0.05). Log‐ratios are shown for each gene in each subject of the three studies.

To find the genes that showed a significant change in expression by the high‐fat challenge in each individual dataset, we analyzed the data per study. An FDR *Q* < 0.05 was used as significant cutoff value (**Figure** 4A). When comparing, the results of these analyses across datasets, 202 genes were found to be differentially expressed in at least two of the three datasets and 67 genes were found to be changed in all three datasets (Figure 4A). **Figure** 5 shows a heatmap of the postprandial gene expression changes in all subjects of the 67 genes that were differentially expressed in all studies. Another method for microarray dataset integration that was applied, involved calculating an overall combined *p*‐value based on *p*‐values of each individual dataset analyzes using Fisher's method. We found 1097 differentially expressed genes due to the high fat challenge at FDR *Q* < 0.05 (Figure 4A).

The third method that we performed was the creation of one merged dataset containing log_2_‐ratios of all subjects from the three datasets. Statistical analysis was performed on this merged dataset, which resulted in 1182 differentially expressed genes by the high‐fat challenge at FDR *Q* < 0.05. Overlap of the genes identified using Fisher's method and the merged dataset analysis was 881 genes, as shown in Figure 4B. Overlap in genes between all comparisons is shown in Figure S1, Supporting Information.

### 3.2 Gene Set Enrichment Analysis

**Figure 6:**
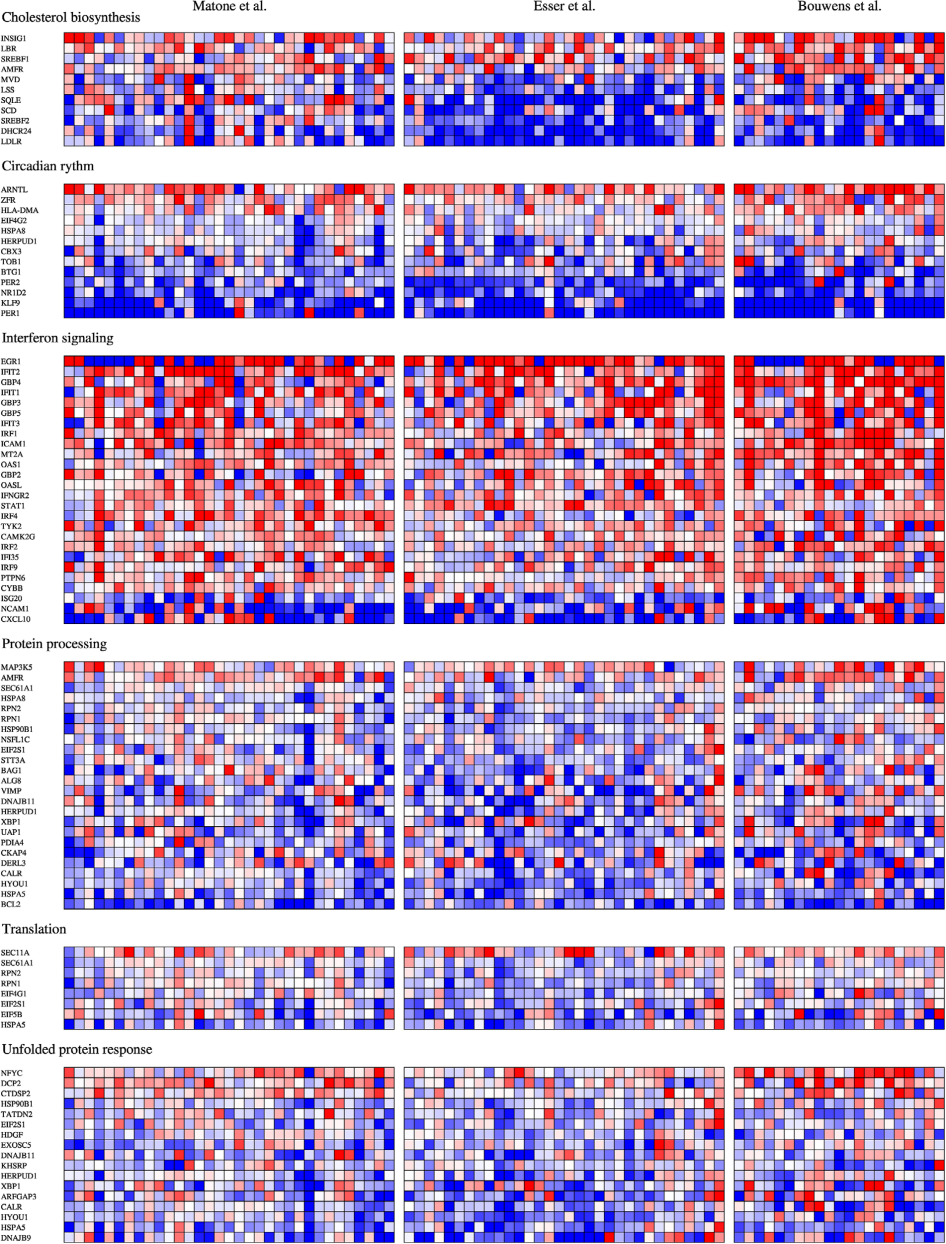
Heatmap of the differentially expressed genes (FDR *Q* < 0.05) in the six differentially expressed gene set clusters (Table 3) in the merged dataset analyses. Log‐ratios are shown for each gene in each subject of the three studies.

To assess which pathways were upregulated by a high‐fat challenge, we performed GSEA on the three separate datasets as well as on the merged dataset. Results are summarized in **Table** 3. Pathways related to circadian rhythm were found to be downregulated in each separate study as well as in the merged dataset. In the merged dataset, pathways related to interferon signaling were upregulated, whereas pathways related to unfolded protein response, protein processing, cholesterol biosynthesis, and translation were downregulated. The individual significant postprandial gene expression changes of genes from these pathways are shown in a heatmap in **Figure** 6.

**Table 3:** Summary of GSEA results

	Bouwens et al.^7^	Matone et al.^8^	Esser et al.^9^	Merged dataset
Interferon signaling	↑	↑		↑
Circadian rhythm	↓	↓	↓	↓
Unfolded protein response		↓	↓	↓
mRNA Splicing			↓	
Protein processing			↓	↓
Cholesterol biosynthesis			↓	↓
Translation		↓		↓
Cell cycle		↓		
Semaphorin processing			↓	
Oxidative phosphorylation		↓		
Toll‐like receptor cascades		↑		
PPAR signaling pathway		↑		

Differentially expressed gene sets were visualized using the Enrichment Map plugin in Cytoscape. Up‐ and downregulated gene sets in separate studies and in the merged dataset are summarized in this table.

## 4 Discussion

We performed a cross‐study integrated analysis of three studies that examined the PBMC gene expression response to a high‐fat challenge using Affymetrix microarrays. Selection of the genes that were differentially expressed in the three separate analyses resulted in 67 differentially expressed genes, which were for a large part involved in circadian pathways. When using different methods, such as the Fishers *p*‐value method and the merged dataset analysis, we observed changes in 1097 and 1182 genes, respectively. Pathways related to interferon signaling were upregulated, whereas pathways related to unfolded protein response, protein processing, cholesterol biosynthesis, and translation were downregulated upon a high‐fat challenge.

Initially, we also included a study (Fat_challenge_tests) that used Illumina beadarrays to measure gene expression in whole blood. As this study showed a large deviation from the others, it was left out in further analysis. We have two potential explanations for the large separation between the Fat_challenge_tests study and the other three studies. First, this study used Illumina BeadArrays, whereas the others used Affymetrix GeneChips. This difference in microarray platform could have induced the bias,^19^ though it has been shown that measurements derived from both platforms show high agreement, especially in genes that are differentially expressed in a comparison.^20^, ^21^ Second, this study also differed in the source of RNA, as whole blood was used whereas PBMCs were investigated in the others three studies. Whole blood contains RNA from all cell types present in blood including granulocytes (neutrophils, eosinophils, basophils), lymphocytes, and monocytes, whereas PBMC RNA samples contain RNA from lymphocytes and monocytes only.^22^ One study compared differences in gene expression profiles in whole blood versus PBMCs in patients with advanced heart failure versus age‐matched controls.^23^They observed a considerable overlap and concordance in gene expression profiles between the two RNA sources, though they also observed differences. Differences in gene expression caused by different blood cell populations were also observed in another study.^22^ In this study, they observed lower S/N ratios and larger variability in whole blood compared to PBMCs. In summary, differences in microarray platforms and cell populations may potentially explain the large separation between the whole blood compared to the PBMC studies.

In the three studies that were further analyzed, we observed consistent up or downregulation of several genes involved in circadian rhythm. In all studies, subject arrived fasted early in the morning and PBMC blood samples were taken just before and 4 or 6 h after the high‐fat challenge consumption. In this regard, the times at which fasting and postprandial samples were taken can be expected to be similar between studies hence explaining the consistent change in genes involved in circadian rhythm. In line with our findings, circadian oscillations in gene expression of PER1 and PER2 have been observed previously in PBMCs of healthy young men.^24^ In addition, PER1 and PER2 gene expression have been shown to peak shortly after awakening 25 or early in the morning,^26^ followed by a gradual decrease in expression in the subsequent hours. These findings coincide with the decrease in gene expression that we observed for these genes 4 and 6 h after consumption of the high‐fat challenge and may thus be independent of the high‐fat challenge.

From studies in rodents, it is clear that many genes, including some involved in metabolic control and immune responses, show a circadian oscillation in gene and protein expression during the day.^27^, ^28^, ^29^ Interestingly, the *IRE1α* pathway, which is part of the unfolded protein response was found to be under circadian control in mice, as observed from a 12 h period rhythmic activation of target genes.^30^ Therefore, the downregulation of pathways related to the unfolded protein response that we observed may potentially be caused by circadian oscillations as well, although studies showing this relationship in humans are lacking. In this study, we do not have a control group available and, we are not able to distinguish between high‐fat challenge‐induced and circadian oscillation changes in gene expression. The individual studies did include multiple challenges and compared differences between them. However, the control challenges differed considerably and could therefore not be used in this analysis. Our analysis of only one of the challenges per study clearly demonstrate the importance of comparing nutritional challenges with different compositions for the identification of actual nutritional challenge‐induced effects on gene expression. However, it should be noted that the nutritional challenges themselves may also influence circadian gene regulation, making it difficult to separate background circadian oscillation from nutrition‐induced changes in gene expression.

Another finding was the upregulation in expression of inflammation‐related genes by the high‐fat challenges. Triglyceride‐rich lipoproteins and lipoprotein remnants are thought to play an important role in mediating postprandial inflammation.^31^ In the postprandial circulation, these lipoproteins may cause the activation of the nuclear factor‐κB transcription factor, either by internalization of these lipoproteins by leukocytes,^32^ or by activating intracellular signaling by binding to CD14 and activation of toll‐like receptor‐4 on the cell surface.^33^ These may be a potential mechanism by which a high‐fat challenge may cause an increase in inflammatory gene expression in PBMCs.

A postprandial downregulation was observed for genes related to cholesterol biosynthesis. Interestingly, this downregulation appears to be mostly attributed to changes in the study by Esser et al. and Bouwens et al., and to a lesser extent the study by Matone et al. The first two studies used a high‐SFA load whereas the Matone et al. study used a high‐MUFA load. In the original study by Esser et al., a high‐MUFA challenge was also included and compared to the high SFA study. Where the SFA challenge reduced expression of genes related to cholesterol biosynthesis, the MUFA challenge did not affect expression of these genes, which is in accordance with our observations in the Matone et al. study. Expression of cholesterol biosynthesis genes is regulated via the *SREBP‐2* transcription factor through a negative feedback system,^34^ suggesting that intracellular cholesterol concentrations may be high after a high‐SFA challenge and not after a high‐MUFA challenge. The cause of this potential cholesterol concentration increase is unclear, as cholesterol biosynthesis gene expression changes appeared to be the largest in the study by Esser et al., even though the high‐fat challenge that was used did not contain cholesterol, unlike the challenge used by Bouwens et al. that did contain cholesterol‐containing butter. Potentially, the higher SFA‐load and the more at‐risk population of the Esser et al. study may have contributed to this effect.

A strong study‐related difference in gene expression was observed (Figure 3A) when comparing log_2_‐intensity values between studies, which is consistent with observations in many previous studies and is a commonly faced problem when combining different batches of microarray data.^19^ Several methods for correction of these batch‐effect have been suggested.^19^, ^35^ Problematic with these approaches is that it is difficult to separate technical batch effects, such as batch effects caused by differences in PBMC collection or lab methodology, from biological effects.^19^ In our studies, differences in subject characteristics (age and BMI) or high‐fat challenge composition are examples of factors that may have caused the batch effects. However, when we examined within‐subject log_2_‐ratios, the study‐related batch effects disappeared, showing a large similarity in the responses to the high‐fat challenge across studies. This is an advantage of the within‐subject design of the studies, as microarrays were performed on samples taken before as well as after the high‐fat challenges in each individual. Furthermore, we found that the overlap in differentially expressed genes between the combined dataset analysis and the Fisher *p*‐value combination method was large, with an overlap of 80%. From these observations, we conclude that the between‐study batch effects are not a major source of variation in our studies. Nevertheless, many differences exist between the three studies, which include differences in study population, challenge composition, time of the postprandial measurement, microarray platform, and laboratory methods. These are all potential sources of variability in our analyzes and may have decreased our power to detect high‐fat challenge‐induced changes in gene expression. Consequently, we may have only picked up the genes and pathways with the strongest changes. In the future, minimizing differences between studies by increased standardization in methods, including the use of a standardized metabolic challenge as described by Stroeve et al.,^2^ the consumption of a standardized low‐fat meal the evening before a challenge test, and using RNA from the same cell sources would greatly enhance the power of these types of integrated analyses. However, the downside of standardization is that the effects of these factors on the response cannot be studied anymore.

To our knowledge, this is the first study that has performed an integrated analysis on microarray datasets from several acute high‐fat challenge intervention studies. We used several integration methods for the combined analysis to identify changes in PBMC gene expression upon a high‐fat challenge. Several differences exist between the studies, such as microarray platform, number and characteristics of subjects, high‐fat challenge composition, laboratory where the analysis took place, and timepoint of the postprandial measurement. Nevertheless, the identified changes are consistent across datasets, demonstrating that this may be a valuable approach that can also be applied in future studies.

Of the used methods, selection of genes that were differentially expressed in all three separate studies was the strictest, resulting in low number of differentially expressed genes that did show the most consistent and robust regulation in all studies. When using different methods, such as the Fisher *p*‐value method and the merged dataset analysis, we observed changes in additional genes and pathways.

In conclusion, the integrated analysis provides added value for the discovery of consistently differentially expressed genes and pathways compared to selecting only those genes and pathways that are identified in all separate studies. Using integrated analyses of PBMC whole genome gene expression responses to high‐fat challenges of studies varying in study population, challenge composition, and research laboratory, we identified a general PBMC whole genome gene expression response to a high fat challenge.

## Conflict of Interest

The authors declare no conflict of interest.

## Supporting information

Supporting Information
