# Supplementary material for: Integrated Analys of High‐Fat Challenge‐Induced Changes in Blood Cell Whole‐Genome Gene Expression
Source: Mol Nutr Food Res. 2019 Sep 30;63(20):1900101. doi: 10.1002/mnfr.201900101 (PMC6856827; doi:10.1002/mnfr.201900101)
Supplement: Supplementary file 1 — Supporting Information [file MNFR-63-na-s001.docx]

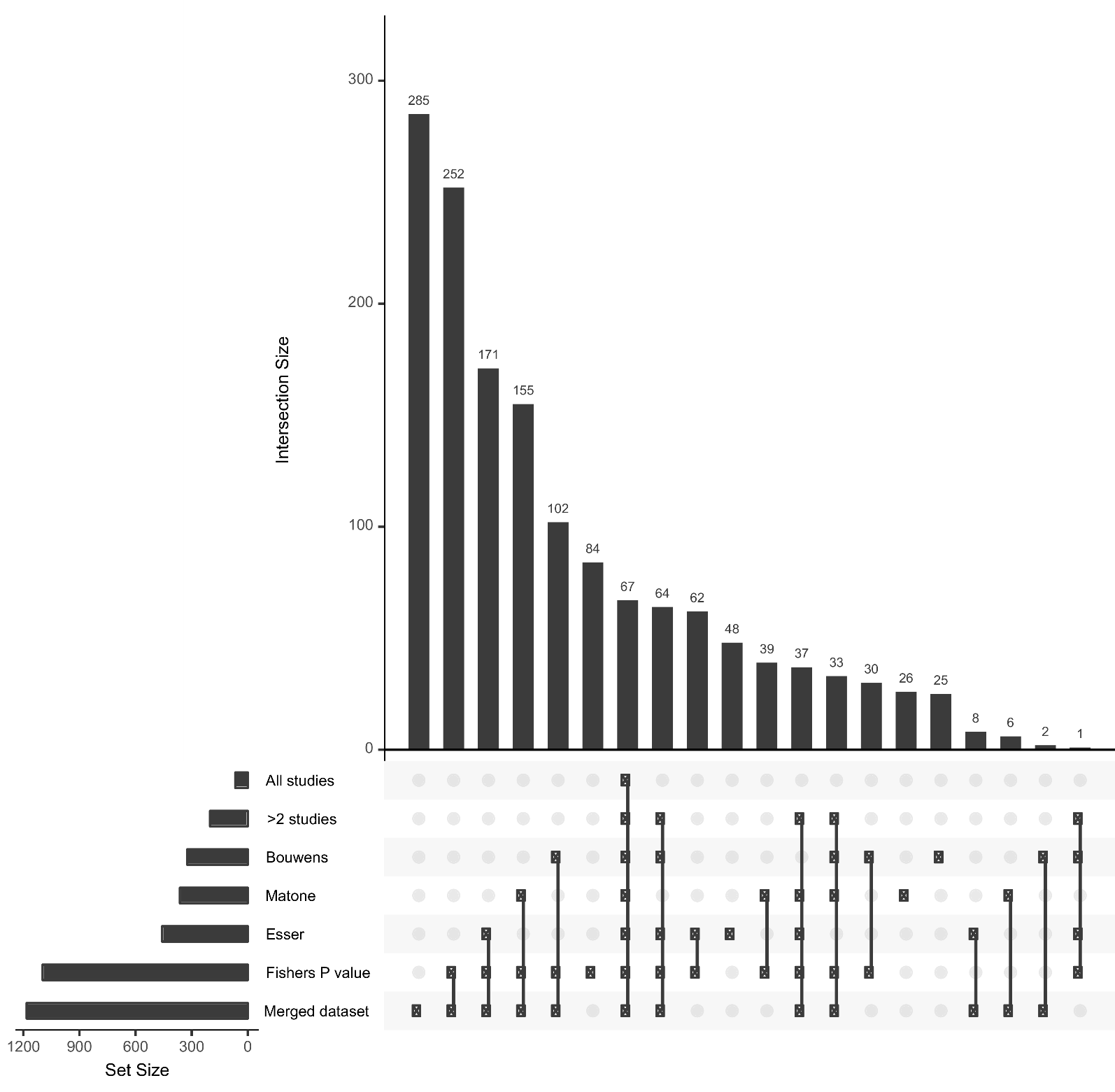


**Supplemental Figure S1.** Overlap in differentially expressed genes in separate studies and in different analysis approaches
